# D-Serine: A Cross Species Review of Safety

**DOI:** 10.3389/fpsyt.2021.726365

**Published:** 2021-08-10

**Authors:** Amir Meftah, Hiroshi Hasegawa, Joshua T. Kantrowitz

**Affiliations:** ^1^College of Physicians and Surgeons, Columbia University, New York City, NY, United States; ^2^New York State Psychiatric Institute, New York City, NY, United States; ^3^Department of Pathophysiology, Tokyo University of Pharmacy and Life Sciences, Tokyo, Japan; ^4^Nathan Kline Institute, Orangeburg, NY, United States

**Keywords:** NMDA–N-methyl-D-aspartate, D-serine, schizophrenia, safety, kidney

## Abstract

**Background:**D-Serine, a direct, full agonist at the D-serine/glycine modulatory site of the N-methyl-D-aspartate-type glutamate receptors (NMDAR), has been assessed as a treatment for multiple psychiatric and neurological conditions. Based on studies in rats, concerns of nephrotoxicity have limited D-serine research in humans, particularly using high doses. A review of D-serine's safety is timely and pertinent, as D-serine remains under active study for schizophrenia, both directly (R61 MH116093) and indirectly through D-amino acid oxidase (DAAO) inhibitors. The principal focus is on nephrotoxicity, but safety in other physiologic and pathophysiologic systems are also reviewed.

**Methods:** Using the search terms “D-serine,” “D-serine and schizophrenia,” “D-serine and safety,” “D-serine and nephrotoxicity” in PubMed, we conducted a systematic review on D-serine safety. D-serine physiology, dose-response and efficacy in clinical studies and dAAO inhibitor safety is also discussed.

**Results:** When D-serine doses >500 mg/kg are used in rats, nephrotoxicity, manifesting as an acute tubular necrosis syndrome, seen within hours of administration is highly common, if not universal. In other species, however, D-serine induced nephrotoxicity has not been reported, even in other rodent species such as mice and rabbits. Even in rats, D--serine related toxicity is dose dependent and reversible; and does not appear to be present in rats at doses producing an acute Cmax of <2,000 nmol/mL. For comparison, the Cmax of D-serine 120 mg/kg, the highest dose tested in humans, is ~500 nmol/mL in acute dosing. Across all published human studies, only one subject has been reported to have abnormal renal values related to D-serine treatment. This abnormality did not clearly map on to the acute tubular necrosis syndrome seen in rats, and fully resolved within a few days of stopping treatment. DAAO inhibitors may be nephroprotective. D-Serine may have a physiologic role in metabolic, extra-pyramidal, cardiac and other systems, but no other clinically significant safety concerns are revealed in the literature.

**Conclusions:** Even before considering human to rat differences in renal physiology, using current FDA guided monitoring paradigms, D-serine appears safe at currently studied maximal doses, with potential safety in combination with DAAO inhibitors.

## Introduction

Glutamate-targeted drugs remain a high priority for the treatment of schizophrenia (1, 2). While no compounds have successfully navigated the difficult process from Phase I to regulatory approval, recent meta-analyses support significant, moderate to large effect size improvements for both schizophrenia symptoms in general, along with specific improvements in negative symptoms, for pooled N-methyl-D-aspartate-type glutamate receptors (NMDAR) modulators adjunctive to antipsychotics compared to placebo (3). In addition to overall improvements in residual psychotic and negative symptoms, glutamatergic based medications have also targeted cognitive deficits (4, 5).

The vast majority of glutamate-based treatment trials have targeted the glycine modulatory site of the NMDAR with natural compounds such as D-serine, glycine, and sarcosine. Recently, the field has seen some successes and some failures with more traditional pharmaceutical glutamatergic treatment trials (5–8). In particular, dose finding, target engagement biomarker work has helped to guide the field (1, 9), allowing an assessment of the ideal doses of the correct compounds to use prior to larger Phase II studies.

The present report focuses on the safety of D-serine, one of the more thoroughly studied NMDAR modulators (3), with a specific focus on potential nephrotoxicity. A review of D-serine's safety is timely and pertinent, as D-serine remains under active study, both directly (10), and indirectly through D-amino acid oxidase (DAAO) inhibitors such as Luvadaxistat (NBI-1065844/TAK-831) and NaBen (sodium benzoate). In addition to a primary focus on D-serine and renal safety, specific topics covered include an overview of D-serine's physiology, efficacy and dose-response in treatment studies, physiology/pathophysiology in other systems and potential metabolic, extra-pyramidal, cardiac, and oncological adverse events and interaction with DAAO inhibitors.

## Methods

Using the search terms “D-serine,” “D-serine and schizophrenia,” “D-serine and safety,” “D-serine and nephrotoxicity” in PubMed, we conducted a systematic review on D-serine safety. The reference lists of articles found were reviewed for additional sources.

## Overview of D-Serine Physiology

Glutamate is the primary excitatory neurotransmitter in the brain, and the NMDAR is the primary glutamate receptor (11, 12). In addition to the primary binding site of glutamate, the NMDAR is modulated by multiple other binding sites. D-Serine is a naturally occurring amino acid that is present in high concentrations in the human brain (13, 14). D-Serine is an NMDAR modulator and a full agonist at the D-serine/glycine site of the NMDAR (15, 16). Binding by D-serine or glycine at this modulatory site is necessary for activation of the NMDAR (11).

D-Serine is the D-isomer of the more common amino acid l-serine. Along with D-aspartate and D-alanine, D-serine is one of the few D-amino acids present in high concentrations in the mammalian brain (or elsewhere in the human body), suggesting an important physiological role (17). The normal source for D-serine in brain appears to be conversion from l-serine, via serine racemase (18, 19). D-serine is converted to back to l-serine only to a limited degree, but in cortical areas with low DAAO, serine racemase appears to degrade D-serine via α/β-elimination of water (20). In general, D-serine is broken down through the action of DAAO (14). In rodents, DAAO is primarily expressed in the cerebellum (21), with only a limited expression in rodent forebrain (22), and thus appears to play a limited role in D-serine degradation in this area (23, 24). DAAO inhibition can modulate hippocampal function in rodents (25). In humans, DAAO is present in both cortical neurons and cerebellar glia (26).

Serine racemase is also present outside the brain (27), but pre-clinical studies suggest that it is less clearly involved in D-serine regulation in the periphery (28). By contrast, DAAO appears to be physiologically active in the periphery, with the largest expression in the cerebellum, small intestine, liver, and kidney (17, 29, 30). Thus, DAAO inhibitors appear to exert their putative therapeutic effects via reduced peripheral degradation of D-serine rather than by direct cortical action.

**Figure 1:**
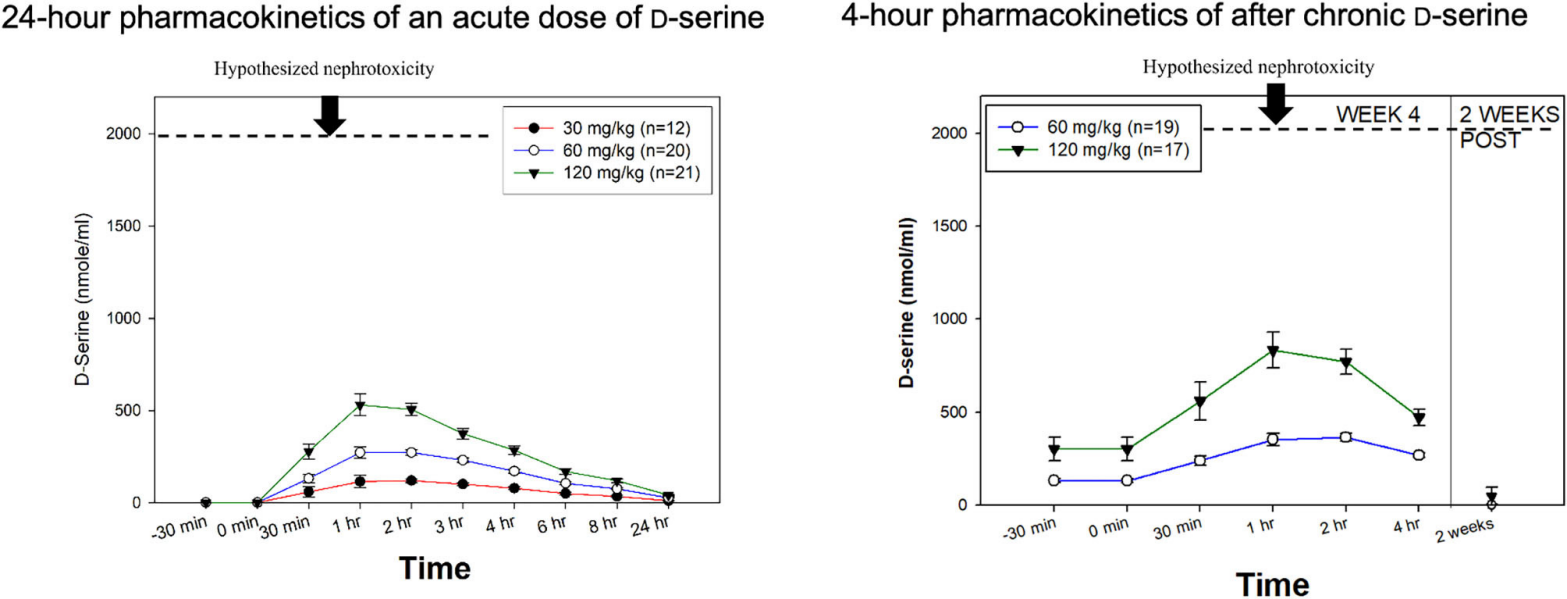
D-serine Pharmacokinetics. **(Left)** 24-h pharmacokinetics of an acute dose of D-serine on day 1 of treatment. **(Right)** 4-h pharmacokinetics after 4 weeks of chronic dosing. In both figures, the hypothesized renal safety level is added based on experiments in rats. Modified from Kantrowitz et al. (31), Hasegawa et al. (108).

In humans, D-serine exhibits linear kinetics (31), with a TMax ~1–2 h following administration (Figure 1, Left) and a t½ of ~3.3 h. The CMax of D-serine is 120.6 ± 34.6, 272.3 ± 62.0, and 530.3 ± 266.8 nmol/ml for the 30, 60, and 120 mg/kg doses, respectively (31). After 4 weeks of daily treatment, linear kinetics continued to be observed, although there may be some modest accumulations (Figure 1, Right).

D-Serine can cross the blood brain barrier, supporting the potential utility as a therapeutic agent (32, 33). Both D-serine and glycine have shown promise in clinical trials, although D-serine may be more pharmacologically potent than glycine (34–38) and is the main NMDAR regulator in cortex. Relevant to its potential as a cognitive enhancer (39), D-serine also has a specific role in long-term potentiation (LTP) and depression (LTD) (40–42), long-term plasticity (43, 44) and synaptogenesis (45). Studies suggest a basal deficit in D-serine in schizophrenia (31, 46), further supporting a role for D-serine as a treatment.

## Use of D-Serine in Treatment Studies: Efficacy and Dose-Dependent Effects

A full listing of the 19 published human studies with D-serine is shown in Table 1. D-Serine has mainly been studied for schizophrenia and related psychotic disorders, but a role for use in tics disorder (61), movement disorders (58), alcohol dependence (64), dementia (65), post-traumatic syndrome disorder (56), and depression (66, 67) have also been proposed and studied.

**Table 1:** Renal safety of D-serine.

**References**	**Active D-serine “n” & diagnosis**	**Dose**	**Renal Abnormalities**
**High dose** **D** **-serine**			
Kantrowitz et al. (47)	20 CHR (prodrome)	60 mg/kg/day for 16 weeks	None
Kantrowitz et al. (4)	21 schizophrenia (Sz)	60 mg/kg single dose × 1week	None
Kantrowitz et al. (48)	16 Sz	60 mg/kg/day for 6 weeks	None
Ermilov et al. (49)	10 Sz	3 g/day for 6 weeks (~45 mg/kg)	None
Kantrowitz et al. (31)	47 Sz	4 week study 12 Sz at 30 mg/kg 19 at 60 mg/kg 16 at 120 mg/kg	1 subject showed 2+ proteinuria without glycosuria after 4 weeks of 120 mg/kg, without change in creatinine
Capitao et al. (50)	20 healthy controls	60 mg/kg single dose	None
Heresco-Levy et al. (51)	1 Sz with anti-NMDAR antibodies	4g for 6 weeks	None
**Low dose** **D** **-serine**			
Tsai et al. (52)	14 Sz	30 mg/kg/day for 6 weeks	None
Tsai et al. (53)	10 Sz	30 mg/kg/day for 6 weeks	None
Heresco-Levy et al. (54)	19 Sz	30 mg/kg/day for 6 weeks	None
Lane et al. (55)	21 Sz	2 g/day for 6 weeks (~30 mg/kg)	None
Heresco-Levy et al. (56)	21 PTSD	30 mg/kg/day for 6 weeks	None
Lane et al. (57)	20 Sz	2 g/day for 6 weeks (~30 mg/kg)	None
Gelfin et al. (58)	8 Parkinson's disease	30 mg/kg/day for 6 weeks	None
D'souza et al. (59)	51 Sz	30 mg/kg/day for 12 weeks	None
Weiser et al. (60)	97 Sz	2 g/day for 16 weeks (~30 mg/kg)	None
Lemmon et al. (61)	9 Tourette's	30 mg/kg/day for 6 weeks	None
Levin et al. (62)	35 healthy controls	2.1 g single dose (~30 mg/kg)	None
Avellar et al. (63)	50 healthy older adults	30 mg/kg single dose	None
**CTP-692**			
Unpublished	244 Sz	12 week study 81 Sz at 1 g 85 at 2 g 78 at 4 g	None

D-Serine was originally reported to be beneficial in schizophrenia based upon studies conducted in Taiwan (52) and Israel (54). A recent meta-analysis of NMDAR modulators in schizophrenia (3) has found specific improvement for D-serine adjunctive to antipsychotics for negative symptoms measured by both the Scale for the Assessment of Negative Symptoms (SANS) (68), with a standardized mean difference (SMD) = −0.56 and the Positive and Negative Symptom Scale (PANSS) negative symptom subscale (69), with a SMD of −0.49. The meta-analysis for D-serine for total PANSS symptoms was not significant (SMD = −0.3). Of note, while a positive trial of the closely related compound D-alanine (70) was included in the meta-analysis, it was not grouped with D-serine as we have done in the past (48). D-Serine's utility as a cognitive enhancer was not evaluated in this meta-analysis.

The majority of human D-serine studies have used a low (30 mg/kg, ~2 g/day) dosage, with a significant, but small effect size improvement (SMD = −0.32) at this dose in meta-analyses (46). This provides proof of concept, but suggests 30 mg/kg may be inadequate to fully engage the NMDAR, as evidenced by larger multi-center studies of 30 mg/kg which failed to separate from placebo (59, 60).

Pre-clinical studies suggest the need for higher doses. As further discussed in the *Renal effects of* *D**-serine* section, rats are especially, and possibly uniquely vulnerable to D-serine induced nephrotoxicity. Thus, pre-clinical behavioral studies need to be completed in mice. In mice, effective doses of D-serine have been in the range of 600–1,000 mg/kg, roughly equivalent to human doses >30 mg/kg (60–120 mg/kg) (71). In other assay systems, numerical reversal of NMDAR antagonist induced (MK-801-induced) hyperactivity in mice was observed at a dose of 600 mg/kg, although significant reduction was not observed until 4,000 mg/kg (72).

Human studies have supported the safety and efficacy of higher dose D-serine, defined as ≥60 mg/kg, ≥4 g/day. An open label dose finding study compared cohorts of 30, 60, and 120 mg/kg/day, finding dose-dependent improvement (31). Significant improvement for total PANSS symptoms was seen at all doses, but specific improvement for both positive and negative symptoms individually was only seen in the 120 mg/kg/day cohort. Similarly, a dose-dependent effect for cognition was seen, finding significantly greater improvement at ≥60 mg/kg vs. Thirty milligram/kilogram dose for the Measurement and Treatment Research to Improve Cognition in Schizophrenia (MCCB) (73) composite (*p* = 0.017). A pharmacodynamic analysis supported a dose effect, finding that higher peak serum levels of D-serine predict greater MCCB scores and improvement on the PANSS in this study, consistent with studies suggesting that basal serum levels of D-serine are related to cognition (74, 75).

The initial double blind studies of high dose D-serine were conducted at a dose of 60 mg/kg, due to caution after a single subject with abnormal renal values at 120 mg/kg (31), as further discussed in the *Renal effects of* *D**-serine* section. A double-blind high dose study in schizophrenia showed significant, large effect size improvements for both total (Cohen's *d* = 0.8) and negative symptoms (*d* = 0.88) (48). Additionally, a nonsignificant, moderate effect size improvement was seen for the MCCB composite (*d* = 0.41) and significant target engagement was seen using mismatch negativity. A high dose study in a clinically high risk (CHR) for schizophrenia group (47) also showed significant improvement in prodromal negative symptoms (*d* = 0.68). Meta-analysis including high dose studies demonstrate moderate to large effect sizes for negative symptoms (3, 48), improving on meta-analysis that only include low dose studies (46).

D-serine as an adjunct to cognitive remediation has been also been proposed (39, 76, 77). One trial used daily low dose D-serine without evidence of efficacy (59), but a trial of 60 mg/kg using an intermittent (once weekly) strategy has shown promising results (4). An ongoing double-blind dose finding study is assessing D-serine doses up to 120 mg/kg (10), using an intermittent dose strategy.

Further evidence for the necessity of testing higher doses of D-serine and related compounds come from the recent negative study of CTP-692 which is a deuterated form of D-serine that reportedly has both less potential renal toxicity and a longer t½ (78). In this publicly reported, but not published study, fixed CTP-692 doses were used, and the highest tested dose was 4 g. Based on publicly available mean weight in kg per dose groups, the highest dose of CTP-692 tested were equivalent to ~45 mg/kg on average (https://ir.concertpharma.com/news-releases/news-release-details/concert-pharmaceuticals-announces-results-ctp-692-phase-2-trial). Thus, even the highest tested doses of CTP-692 may have been too low, which may have contributed to the negative study.

## Renal Effects of D-Serine

In addition to their importance in the brain, NMDAR are found throughout the body, including the kidney, where they play a diverse, if not fully elucidated role (79–81). D-Serine is also found in the kidney, with a potential physiological role (80).

The potential risk of D-serine induced nephrotoxicity has been described since the 1940's (82–84), primarily based on studies in rats, and classically leads to a reversible acute necrosis, termed acute tubular necrosis. Pathological changes are present within 1 to 2 h post D-serine administration, and are generally limited to necrotic changes of the straight segment of the proximal tubule (85–87), which is the primary site of D-serine reabsorption (88). The earliest changes are pronounced eosinophilia in the straight proximal tubules (87). Concurrently, acute increases in urine volume, glucosuria, proteinuria, and aminoaciduria, including D-serine are seen (85, 86), while sodium and potassium excretion remains stable. D-Serine excretion peaks within the first 8 h post dose (87). Other specific findings include granular (muddy) casts seen on urinalysis.

Despite these acute pathological changes, D-serine induced nephrotoxicity appears to be fully reversible (85), even in rats. Urine values of protein, glucose and amino acids begin to normalize 24–48 h after the last dose of D-serine and by 120 h post dose, largely return to normal (87). Pathological changes also completely resolve within this timeframe, with complete regrowth of new epithelium in tubules and renal tubular basophilia (87).

In addition to being reversible, D-serine induced nephrotoxicity has only been observed in rats. In other species, including other rodents, D-serine induced nephrotoxicity has not been reported. Tested species include guinea pigs, rabbits, and mice (84), along with dogs, hamsters, and gerbils (89). Most importantly for the treatment of psychiatric disease in humans, is the lack of evidence for D-serine induced nephrotoxicity in humans (Table 1). Even in rats, this heightened risk to D-serine does not appear to occur during “normal,” physiological levels of D-serine.

The etiology for the isolated risk to rats as compared to other species is not completely clear, but appears to be due to both higher reabsorption of D-serine by rat kidneys compared to other species and differences in DAAO function. The presence of enhanced reabsorption is apparent from the low levels of D-serine in rat urine relative to that of other species, such humans and dogs, despite relatively similar serum levels (90). Moreover, nephrotoxicity during exogenous D-serine administration may be related to oxidative stress from the increased DAAO breakdown of D-serine (29, 91–93). DAAO is localized in pars recta of the kidney, where D-serine (94, 95) is primarily reabsorbed and the focal point of damage during D-serine nephrotoxicity. While levels of DAAO in rats do not appear to be quantitatively different than in other species (96), rat DAAO may be less efficient, which may compound the risk of nephrotoxicity due to hyperfunction during periods of excess D-serine (97). Relatedly, reducing DAAO activity through DAAO knockouts or concurrent DAAO inhibitors may be nephroprotective to excess D-serine (see *d**AAO clinical and safety* section). Finally, studies also suggest that rats may have a higher capacity of utilizing D-amino acids (29) and that NMDAR may be directly involved in producing nephrotoxicity (98).

By contrast to rats, in most other species, including humans, D-serine is not actively reabsorbed (90, 99, 100), as evidenced by relatively higher D-serine urine levels in humans compared to rats of D-serine under physiological conditions (90). In humans, D-serine does not accumulate in serum under physiologic conditions, other than in people (101, 102) or mice (103) with pre-existing renal impairment. Under these pathological conditions, D-serine may be a biomarker of renal disease or recovery in humans (104–107), rising or falling in proportion to creatinine. However, there does not appear to be a causal link between D-serine and renal impairment.

Even in rats, D-serine nephrotoxicity appears to be dose related. The initial rat toxicity studies used doses of 750–1,000 mg/kg (83, 85, 86), and in doses ≥500 mg/kg, nephrotoxicity after D-serine treatment appears to be very common, if not universal in rats. Similar to mice (71), however, the oral dose to serum concentration ratio does not appear to follow a 1:1 ratio in rats compared to humans, complicating direct translational studies.

Recently, the pharmacokinetics and toxicokinetics of D-serine in rats was systematically studied (108), potentially allowing for a more direct rat to human comparison. In this study, five intraperitoneal doses were tested, 0.6, 1.2, 1.8, 2.4, and 4.8 mmol/kg. Based on an assumption of linear pharmacokinetics and a comparison with human studies (31), the 1.8 mmol/kg rat dose is thought to be approximately equivalent to an oral human dose of 450 mg/kg, ~3× the highest tested human dose. No nephrotoxicity was observed at either 6 or 24 h post dose at the 0.6 or 1.2 mmol/kg doses. Beginning at 1.8 mmol/kg, significant dose dependent elevations are seen for urine protein and glucose compared to the 0.6 mmol/kg dose at 6 h and for serum creatine from baseline at 24 h. Toxicity was also seen at higher doses (2.4 and 4.8 mmol/kg).

A Cmax of ~2,000 nmol/mL was the dividing line between safety and nephrotoxicity in this study, which was achieved with the 450 mg/kg equivalent dose (1.8 mmol/kg) (see Figure 1). Additional support for a dose response for toxicity in rats was shown in a study in which doses ≤to 250 mg/kg were safe, while 500 mg/kg produced the expected nephrotoxicity (87). Other studies have reported toxicity at 400 mg/kg (92). For comparison, the single dose Cmax of 120 mg/kg, the highest dose tested in humans, was 530.3±266.8 nmol/mL in acute dosing (31). After 4 weeks of chronic dosing, there was some accumulation, but the Cmax remained well-below 2,000 nmol/mL (~800 nmol/mL). We are aware of one study suggesting that extremely large doses of D-serine can induce nephrotoxicity in a cell culture of human renal tubular cells (109). However, this study used D-serine concentrations of 10 to 20 mM, which are 20 to 40 times greater than the Cmax of 120 mg/kg (0.5 mM or ~500 μM).

Nineteen human trials have been published or publicly presented with D-serine or the closely related compound of CTP-692 (Table 1), including 490 subjects receiving D-serine with treatment durations ranging from single doses to 16 weeks of daily dosing and 244 patients on CTP-692. One hundred twenty-two subjects received high dose D-serine (>30 mg/kg), including 16 patients receiving 120 mg/kg for 4 weeks. Seventy-Eight subjects received high dose CTP-692, defined as 4 g per day. Across all studies, only one subject was reported to have abnormal renal values related to D-serine treatment (31). Overall, this 1 case represents 0.2% of all D-serine treated subjects, <1% of subjects treated with daily high dose D-serine and one of 16 (6.3%) of subjects treated with 120 mg/kg daily. Several mild out of range renal values were noted in the CHR study (47). No renal adverse effects were reported in the CTP-692 study.

The single abnormality at 120 mg/kg occurred in a subject after receiving 4 weeks of the 120 mg/kg dose. This abnormality was considered mild in that it involved only an increase in protein (2+ by dipstick) without granular casts or an accompanying increase in glycosuria, change in creatinine level or other clinical correlates of renal dysfunction, and fully resolved within a few days of stopping treatment. Thus, this abnormality does not clearly map on to the acute nephrotoxicity syndrome seen in rats. Under our current FDA approved safety monitoring criteria, fully described in the *Recommendations for monitoring during clinical* *d**-serine studies* section, this abnormality would not have been considered a serious adverse event (SAE).

## D-Serine and the Pancreas and Metabolism

Moving beyond the brain and the kidney, D-Serine may play a physiologic role in both appetite and insulin regulation in the pancreas, which is of potential clinical relevance since many antipsychotics are associated with clinically significant weight gain and metabolic disturbances (110, 111). D-Serine appears to be elevated in pre-clinical mice models of diabetes, but this seems to be an effect, and not a contributing cause of diabetes *in vivo* (112, 113). As recently reviewed (114, 115), functional NMDAR are found in pancreatic islets and β-cells, which regulate insulin release. The role of NMDAR in the pancreas is complex, with some studies suggesting that NMDAR antagonism would be therapeutic, and some suggesting the opposite. A similarly unclear role is found for D-serine itself, and D-serine has been studied both as a potential treatment for metabolic disorders and for adverse effects.

Part of the complexity and lack of clarity of the NMDAR and D-serine's role in glucose homeostasis stems from the varied dosages that were used in pre-clinical experiments. Under physiologic conditions, D-serine appears to activate pancreatic NMDAR to stimulate ß-cell and potentiate insulin release (116). At higher, non-physiologic doses, D-serine may lead to toxicity due to NMDAR internalization, reducing ß-cell activity, and reduced insulin release. Serine racemase is present and active in the pancreas (117), and helps regulate insulin secretion (118), further suggesting a role for D-serine. By contrast, a recent study (119) suggests that large doses chronic D-serine supplementation results in both reduced high fat diet intake and impaired insulin secretion in mice. In this study, mice received 10 g of D-serine/L of water, and assuming a 25 g mouse drinks 5 mL of water/day (120), the doses required to impair insulin secretion were large (~2,000 mg/kg), and thus may be of questionable clinical relevance.

Other studies (121, 122) have also supported a dose dependent role for D-serine suppressing intake of high preference (high-fat) food, suggesting potential utility in modulating obesity. In these studies, an appetite suppressant effect was seen at D-serine >1.5 g/kg per day, but not at lower doses. The largest doses studied in humans are ~10× smaller (120 mg/kg), limiting the translation of these findings to human studies.

Two recently published human studies assessing D-serine's role in monitoring diabetes have shown inconsistent results. Across one study with 96 women with gestational diabetes and 96 with normal glucose tolerance, serine was significantly higher in the gestational diabetes cohort (123). By contrast, in a separate study of 1,623 non-diabetic subjects (124), the opposite result was seen, as lower serine levels were predictive of impaired glucose tolerance. In both studies, we note that the term serine is used, and it is unclear if the measurements were of D-serine, l-serine or a combination. In published studies, no clinically relevant weight gain or metabolic alterations have been reported in clinical studies of D-serine (Table 2).

**Table 2:** Adverse events reported in d-serine trials^a^.

**Adverse event**	**Total *n***	**D-serine (%)**	**Placebo (%)**	**Risk ratio (95% CI); *p*-value**
Abdominal discomfort	31	0	5.9	0.40 (0.02, 9.12); 0.57
Anxiety	84	4.9	9.3	0.52 (0.10, 2.70); 0.44
Constipation	115	7.3	0	3.93 (0.66, 23.25); 0.13
Depression	44	4.8	4.3	1.10 (0.07, 16.43); 0.95
Diarrhea	31	7.1	0	3.60 (0.16, 82.05); 0.42
Dizziness	192	15.8	26.5	0.61 (0.32, 1.18); 0.14
Dry mouth	149	5.4	0	9.12 (0.50, 166.46); 0.14
Fatigability	84	22.0	23.2	0.96 (0.19, 4.74); 0.96
Headache	149	17.6	38.7	0.45 (0.26, 0.80); 0.007
Nausea	31	14.3	0	6.00 (0.31, 115.56); 0.24
Palpitation	84	29.3	27.9	1.06 (0.45, 2.48); 0.89
Salivation	44	14.3	8.7	1.64 (0.30, 8.89); 0.56
Sexual dysfunction	26	7.7	0	3.00 (0.13, 67.51); 0.49
Sleep disturbance	115	21.8	20	1.07 (0.53, 2.18); 0.85
Weight gain	84	43.9	44.2	1.05 (0.69, 1.59); 0.81
Weight loss	84	7.3	4.7	1.36 (0.16, 11.68); 0.78

a *Modified from Goh (3)*.

## D-Serine and the Endocrine System

Aside from the brain, the kidney and the pancreas, D-serine has been most thoroughly studied in endocrine systems. As recently reviewed (17), D-serine is detected *in vivo* in multiple endocrine glands, including the hypothalamus, pituitary, pineal, thyroid, adrenals, ovary, and testes. However, levels of D-serine in the endocrine organs are lower than those in the CNS, and the physiological role of D-serine in most endocrine organs is unclear.

A role for a regulation of sleep has been reported for both glycine and D-serine, following up on small clinical studies of glycine (125). In a pre-clinical study, improved sleep was seen with direct injection of either glycine or D-serine into the suprachiasmatic nucleus of the hypothalamus (126). D-Serine may also be involved in activating the NMDAR in the corpus cavernosum, suggesting a possible role in treating impotence (127).

## D-Serine and Extrapyramidal Effects

Antipsychotics are associated with varying levels of extrapyramidal motor side-effects (EPS) (110), such as Parkinson's like motor disturbances, tremor and dystonia. While one of the clearest advantages of many second generation antipsychotics is a relatively reduced incidence of EPS and other movement disorders such as tardive dyskinesia (TD) (128), both remain a clinically significant issue for many schizophrenia patients.

Antipsychotics likely cause EPS via dopamine type 2 receptor blockade in the striatum. In a pre-clinical mouse study (129), both D-serine (300 mg/kg) and sodium benzoate (600 mg/kg) administered intraperitoneally attenuated haloperidol induced bradykinesia. D-serine showed a *U*-shaped curve for attenuation, as no effects were seen for 100 or 1,000 mg/kg doses. Our pre-clinical studies with mice (71), suggest a comparable mice dose of approximately 100 mg/kg for the 60 mg/kg clinical dose. In this study, D-cycloserine, which acts as an agonist at the D-serine/glycine site of the NMDAR, but a ketamine like antagonist at higher doses (130–132), also attenuated haloperidol induced bradykinesia at doses up to 30 mg/kg, which likely is in the NMDAR agonist range.

Two clinical studies with D-serine have suggested improvement in antipsychotic induced EPS and/or TD in schizophrenia patients (31, 54). One small study of 8 patients suggested efficacy of low dose D-serine for both the behavioral and motor symptoms of Parkinson's disease (58). A double blind study of high dose D-serine did not find a significant benefit for EPS (48).

Amyotrophic lateral sclerosis (ALS) is a fatal neurodegenerative disorder involving an extensive loss of motor neurons, and some familial and sporadic cases have been associated with D-serine metabolism. Specifically, mutations of DAAO have been reported (133), which are associated with pre-clinical and clinical increases of D-serine (30, 134). One recent study found elevated plasma levels of D-serine in ~40% of ALS patients compared to healthy controls (135). Based on publicly presented, but unpublished observations in studies conducted to support our IND, there is no evidence of D-serine accumulation in motor neurons (71), and there has been no evidence of motor adverse events in human studies.

## D-Serine, the Liver and the Gastrointestinal Tract

D-Serine is cleared almost exclusively by the kidney, and is not metabolized by hepatic P450 enzymes. DAAO is present in the liver, and may contribute to D-serine degradation (136). The pre-clinical literature of D-serine's effects on the liver are sparse, but early experiments did not find evidence of a D-serine specific hepatoxic effect in rats using known nephrotoxic doses (1,000 mg/kg) (83). One study using extremely large doses of D-serine (20 mM) was hepatotoxic to *in vitro* rat liver cells and mitochondria, producing oxidative stress and swelling (137). In clinical studies, mild, asymptomatic transaminitis has been reported in two subjects receiving daily 120 mg/kg (31). Only one of the subjects had liver function tests (LFTs) >2× upper normal range. This mild transaminitis resolved completely after D-serine discontinuation for both patients, and may have been related to the recent administration of the hepatitis vaccine in the patient with the larger elevations, which in rare cases can give rise to elevated liver enzymes (http://vaers.hhs.gov).

In mice, D-serine has shown promise as a treatment and prophylaxis for inflammatory bowel disease (138), albeit at high doses, >1.5 g/kg per day. Finally, D-serine may be involved in lower esophageal sphincter contraction (139), with unclear clinical relevance. D-Serine has not been associated with elevated rates of gastrointestinal adverse events in clinical studies (Table 2).

## D-Serine and the Cardiovascular System

As recently reviewed, NMDAR are also present in cardiac and vascular tissue (140), and activation of these peripheral NMDAR *in vitro* can lead to tachycardia and hypertension (141). While there is no known physiologic role for D-serine in the heart, D-serine could theoretically lead to increased cardiovascular tone by activating NMDAR. By contrast, the NMDAR antagonist ketamine, consistently produces tachycardia and hypertension in clinical studies (1). While direct application of ketamine on *in vitro* cardiac tissue induces bradycardia (142), the tachycardic/hypertensive effects of *in vivo* ketamine are mediated through brain, with evidence for both centrally mediated top-down control (143–145) and direct effects on the baroreflex in the nucleus tractus solitarii (NTS) in the brainstem (medulla) (146–149). No clinically relevant cardiovascular effects have been reported in clinical studies of D-serine.

## D-Serine and Cancer

As recently reviewed (150), D-amino acids may be elevated in some cancers. D-Serine does not appear to be a causal factor in tumorigenesis, but there may be increased reuptake of D-serine by some cancer cells, particularly in high glucose environments (151). Alternatively, D-amino acids may be useful for the treatment of some cancers (152–154).

## DAAO Inhibitor Clinical Studies and Safety

DAAO-inhibitors have been proposed as a treatment for schizophrenia, functioning in a similar way to a selective serotonin reuptake inhibitor (SSRI) by increasing D-serine levels indirectly. Several DAAO-inhibitors are in development, including luvadaxistat and sodium benzoate. Sodium benzoate has shown efficacy in several, but not all published studies (155–158), and is being actively developed by SyneuRx International (NCT02261519). Luvadaxistat is under development by a partnership between Takeda and Neurocrine (159), and showed preliminary efficacy for cognitive outcomes in publicly presented, but unpublished results.

Although DAAO-inhibitors raise the levels of D-serine and increased DAAO activity may be contributory to nephrotoxicity in rats (91–93), pre-clinical studies suggest that DAAO-inhibitors may protect against D-serine induced nephrotoxicity (29, 160). In a study of rats without functional DAAO activity, D-serine 800 mg/kg did not cause renal damage (29). Furthermore, administration of D-propargylglycine, which is known to cause nephrotoxicity through DAAO (161), also did not cause renal damage in the DAAO knockout rats. By contrast, both D-serine and D-propargylglycine led to the expected nephrotoxicity in the control rats with normal DAAO.

Direct evidence that DAAO-inhibitors are nephroprotective has also been demonstrated in rats (160). In this study, rats were given D-serine 500 mg/kg 1 h after receiving one of 4 doses of sodium benzoate (125, 250, 500, or 750 mg/kg). A dose dependent nephroprotective effect was seen with pretreatment with sodium benzoate 500 mg/kg or greater. The protective effects were most apparent in the first urinalysis samples several hours after D-serine. Pathological samples after 24 h with and without sodium benzoate showed nephrotoxic changes, but sodium benzoate appeared to attenuate these changes as compared to the D-serine alone samples. There has been no reported renal toxicity reported in clinical studies of DAAO-inhibitors. Taken together, these studies support the safety of potential combined D-serine + DAAO-inhibitor studies, which have shown promise pre-clinically (162–164).

## Adverse Events in Clinical Studies of D-Serine

In Table 2, we present a summary of adverse events in published trials of D-serine, modified from a similar table in a meta-analysis of NMDAR trials in schizophrenia (3). As in the meta-analysis, the present report uses the total of all subjects in which an adverse event is reported as the total potentially affected, rather than the total number in all studies. This allows for a more conservative estimate of the rates of an adverse event. The downside to the analysis is that adverse events were not systematically reported in most of these studies, and the overall n is small. Noting these caveats, in these studies, the only adverse event reported at a significantly different rate than placebo is headache, finding a significantly lower rate of headaches in the of D-serine group.

## Recommendations for Monitoring During Clinical D-Serine Studies

In our FDA-monitored studies, we monitor for safety as follows. Routine safety laboratory measures, including a chemistry with serum creatinine and LFTs, a complete blood count and a urinalysis with microscopics, are obtained at screening. Vitals and ECGs are also obtained. No subjects with baseline renal impairment, as evidenced by an estimated glomerular filtration rate (eGFR) <60 or clinically significant abnormal laboratories are enrolled.

During the study, potential nephrotoxicity is monitored through serum chemistry and urine microscopic examination looking for evidence of active sediment (e.g., casts), proteinuria or glycosuria, as per FDA guidance.

After randomization, we monitor as follows:

(a) Urinalysis with microscopics and chemistry biweekly for daily studies or after each dose for intermittent treatment.
(b) Immediately discontinue D-serine for unexplained serum creatinine increase >0.3 mg/dL over the pre-study value or for >1 granular or muddy casts. Treat as SAE possibly related to study medication. Repeat until clear × 2 to demonstrate reversibility.
(c) Hold D-serine for >1 hyaline casts, and repeat lab. Ask subject to eat more salt and drink more water. If absent on repeat, reinstate D-serine and treat as adverse event (AE). If present on repeat, continue to hold D-serine and repeat lab once again. If still present on second repeat, discontinue D-serine and treat as SAE possibly related to study medication. Repeat until clear × 2 to demonstrate reversibility.
(d) Hold D-serine for proteinuria >100 mg/dl or unexplained glucose >250 g/dl (both equivalent to 2+). If absent on repeat, resume D-serine and treat as AE. If still present on repeat, discontinue D-serine. Repeat until clear x 2 to demonstrate reversibility. This would be treated as SAE possibly related to study medication. Unexplained glycosuria is defined as increased urine glucose in absence of corresponding increase in serum glucose levels, in patients without glycosuria at baseline.
(e) Continue D-serine for proteinuria>30 but<100 mg/dl (1+), or unexplained glycosuria (>100 but <250 g/dl) but repeat. If absent on repeat, continue D-serine and treat as AE. If still present on repeat, hold D-serine and repeat once more. If absent on repeat, resume D-serine and treat as AE. If still present on second repeat, discontinue D-serine and treat as SAE possibly related to study medication. Repeat until clear × 2 to demonstrate reversibility.
(f) For other kidney related measures (e.g., ketones, bilirubin, WBC, RBC, bacteria, crystals), repeat, but no need to discontinue even if present on repeat, since unlikely to be D-serine related. Manage in consultation with medical specialist.
(g) Contaminated samples (hemolyzed/non-clean catch/menstruation) will be repeated.

## Conclusions

Schizophrenia remains a difficult to treat illness, with a large majority of patients not responding completely to FDA approved antipsychotics. D-Serine appears efficacious in schizophrenia, especially in high doses (≥60 mg/kg). Our literature review supports that D-serine is safe and well-tolerated in people without pre-existing renal dysfunction. While there is no evidence of D-serine being nephrotoxic in humans, we require that people with pre-existing renal dysfunction (GFR <60) be excluded from clinical studies.

Thus far, 120 mg/kg is the highest D-serine dose tested in human studies, but animal studies suggest that even higher doses may be required for optimal target engagement. In this review, we have taken a conservative approach to interspecies dose equivalences, but note that standard mouse to human conversions of 12.3 to 1 have been proposed in the literature (165). Nevertheless, even before considering human to rodent differences in physiology, the literature supports that D-serine has potential safety at doses even higher than 120 mg/kg. Ongoing dose-response studies are assessing the safety and efficacy of doses up to 120 mg/kg, and future work is needed to explore the possibility of even higher doses or combined D-serine + DAAO-inhibitor studies.

## Author Contributions

JK and AM: substantial contributions to conception and design. JK, AM, and HH: drafting of the manuscript and critical revision of the manuscript for important intellectual content. All authors reviewed the final submission and gave final approval of the submitted version.

## Conflict of Interest

The authors declare that the research was conducted in the absence of any commercial or financial relationships that could be construed as a potential conflict of interest.

## Publisher's Note

All claims expressed in this article are solely those of the authors and do not necessarily represent those of their affiliated organizations, or those of the publisher, the editors and the reviewers. Any product that may be evaluated in this article, or claim that may be made by its manufacturer, is not guaranteed or endorsed by the publisher.
